# Predicting Weight Control Intentions: An Extended Model of Goal-Directed Behavior

**DOI:** 10.3390/ijerph22040600

**Published:** 2025-04-11

**Authors:** Hyun Ju Yun, Yumi Jang, Jee Hye Lee

**Affiliations:** Department of Food Science and Nutrition, University of Ulsan, Ulsan 44610, Republic of Korea; goidcat@ulsan.ac.kr (H.J.Y.); yjang@ulsan.ac.kr (Y.J.)

**Keywords:** weight control, extended model of goal-directed behavior (MGB), mental health, intention, desire

## Abstract

This study aimed to develop an extended model of goal-directed behavior (MGB) to more thoroughly explain the intention of adults to control body weight. The extended model integrates the crucial constructs and a newly added construct, mental health, as a formative second-order factor in the weight control context into the original MGB. An online survey was conducted with 239 undergraduate students, who responded to the constructs of attitude, subjective norms, anticipated emotions, desire, and mental health. A descriptive analysis was conducted, and the fit of the proposed research model was verified using structural equation modeling. The results showed that the known constructs of attitude, subjective norm, and anticipated emotions were critical predictors of desire in the context of weight control. In addition, desire influenced intention to control weight. Furthermore, mental health had a significant causal relationship with the variables in the extended goal-directed behavior. It broadens the weight control literature by emphasizing the role of affective factors in weight control behavior, expanding the MGB theory with mental health, and providing empirical evidence for an effective weight control intervention program.

## 1. Introduction

There is a significant interest in weight control among young Koreans, not only for health maintenance, but also for esthetic reasons, with various weight control methods actively being pursued [1,2,3]. Despite the increased focus on weight, the prevalence of obesity is expected to increase from 2020 to 2030 [4]. Approximately 36.3% and 23.9% of Korean adults demonstrated abdominal and overall obesity, respectively, which is higher than the world average of 13% [5]. Some obese people struggle with weight loss because of difficulties in maintaining a healthy diet and regular physical activity [6], and lifestyle-based weight reduction centered around change is challenging [7]. Thus, previous research emphasized the critical role of motivational variables in health-related behaviors [6,8]. An investigation of the predictors influencing intention for weight control is necessary for effective weight management and can provide basic data for weight control intervention and education programs [9].

Several theories have been proposed to explain health-related behaviors, including the health belief model, theory of reasoned action, theory of planned behavior, and the integrated behavioral model [10,11]. The theory of planned behavior (TPB) has been used to explain individual health-related behaviors and has been widely applied in predicting individual behaviors such as healthy eating habits, exercise, and weight loss behavior. The TPB is an expansion of the theory of reasoned action, which suggests that individual attitudes and subjective norms influence human behavioral prediction [12,13]. The TPB, proposed by Ajzen, suggests that attitudes, subjective norms (social pressure), and perceived behavioral control (an individual’s perceived ability to control their behavior) influence the intention to engage in a particular behavior [14]. The theory posits that the stronger are these factors, the higher will be the intention to act, ultimately leading to actual behavior. Armitage and Conner later criticized the low (29%) variance of predictors in behavior of the TPB and questioned its sufficiency [15].

In 2001, Perugini and Bagozzi introduced the model of goal-directed behavior (MGB) to overcome the shortcomings of the theory of reasoned action because the TPB fails to consider individual emotional aspects, desire, and past behavior and inadequately represents relationships between constituent concepts [16]. To improve individual behavioral intention, the MGB includes emotional components such as positive and negative anticipated emotions, motivational components like desire, and frequency and recency components like past behavior [16]. The essential constituent variables in the MGB models are attitudes, subjective norms, perceived behavior control, positive and negative anticipated emotion, desire, past behaviors, behavioral intention, and behavior. The concepts of each variable are as follows. The term “attitude” refers to an overall preference for behavior and entails favorable or unfavorable responses toward an object [12,17]. Subjective norm is the perceived social pressure regarding performance of a specific behavior [18]. Perugini and Bagozzi suggested that the perception of positive evaluations from significant others regarding one’s behavior imparts an increase in motivation for that behavior [16]. Conversely, when significant others hold negative attitudes toward the behavior, motivation for the behavior decreases.

Anticipated emotion is the feeling experienced in uncertain situations before achieving a goal [19]. In detail, positive anticipated emotion is the expected positive feelings linked to reaching a goal, whereas negative anticipated emotion refers to the expected negative feelings associated with failing to achieve a goal [16]. Desire refers to the emotional motivation individuals have toward a goal and is a key driver of action; it serves as both a mediating variable prompting action and a crucial driver of behavior [16]. Desire plays a pivotal role, arising when an individual aims for specific outcomes, directly explaining behavioral intentions and indirectly influencing the intentions toward independent variables of planned behavior theory [20].

The MGB is one of the most reliable models for predicting health-related behavior [21,22]. Previous studies that have applied the MGB theory in the health field indicated that personal attitudes, values, and emotions positively influence desire toward behavioral intentions [23,24]. Weight control has been applied in the theory of planned behavior but not yet integrated into MGB.

In this study, mental health is considered in the MGB to better comprehend the motivational drivers underlying behavioral change toward weight control. Previous evidence demonstrated that mental health is closely associated with behavior [25]. For instance, depressive symptoms were reported to relate to health behavior such as a higher body mass index (BMI) or lower physical activity [26,27]. Negative mental states such as severe depression and anxiety interfere with the ability to control actions [28,29]. Women vulnerable to poor mental health experienced higher levels of anxiety, depression, and loneliness, along with unhealthy behaviors like increased smoking and screen time and decreased physical activity [30]. However, previous studies that incorporated mental health within the context of weight control have been limited. This study investigated mental health as a critical factor for predicting the intention to control weight.

**Figure 1:**
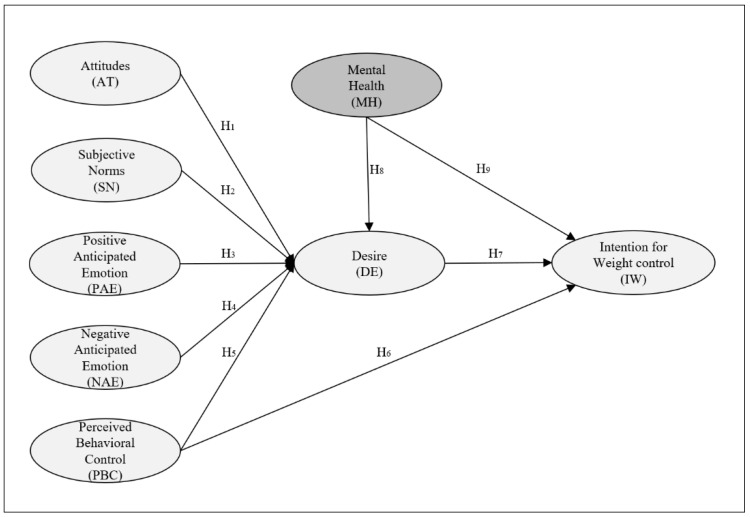
A proposed research model. Note 1. The shaded area indicates the newly added constructs in this study.

Building on the theoretical background, the present study aimed to apply the extended MGB to explain weight control intentions of undergraduates; this analysis is the first empirical examination of extended MGB in a weight control context. The directions of this study were as follows: (1) The extended MGB was employed, incorporating attitude, subjective norms, perceived behavioral control, anticipated emotions, desire, and behavioral intention. (2) Mental health was introduced as an antecedent to desire and behavioral intention. Therefore, this study extends the original MGB in the weight control context by incorporating mental health to elucidate the decision-making processes of weight control behavior (Figure 1).

The hypotheses proposed in this study are as follows.

**H1.**  *Attitude toward weight control has a positive influence on desire for weight control.*

**H2.**  *Subjective norms for weight control have a positive influence on desire for weight control*.

**H3.**  *Positive anticipated emotion regarding weight control has a positive influence on desire for weight control*.

**H4.**  *Negative anticipated emotion regarding weight control has a negative influence on desire for weight control*.

**H5.**  *Perceived control over weight control has a positive influence on desire for weight control*.

**H6.**  *Perceived control over weight control has a positive influence on intention for weight control*.

**H7.**  *Desire for weight control has a positive influence on intention for weight control*.

**H8.**  *Mental health has a positive influence on desire for weight control*.

**H9.**  *Mental health has a positive influence on intention for weight control*.

## 2. Methods

### 2.1. Measurements

All scales used in this study were obtained from the literature. The questionnaire comprised two sections. One part asks questions on general demographic characteristics including gender, grade, BMI, and weight-related questions (e.g., satisfaction with body shape, reasons for weight control, and type of desired weight control). The BMI classifications are based on the criteria provided by Korean Society for the Study of Obesity (2014): underweight (BMI < 18.5 kg/m^2^), normal weight (18.5–22.9 kg/m^2^), overweight (BMI ≥ 23 kg/m^2^), and obesity (BMI ≥ 25 kg/m^2^).

The second part asks questions on the construct, including attitudes, subjective norm, positive anticipated emotion, negative anticipated emotion, perceived control, desire, and intention (Table 1). The questions (Table 2) were adopted from the MGB theory [16,18,31] using a 7-point Likert scale (1 = not at all, 7 = very much). Additionally, mental health consists of five dimensions of obsession-compulsivity, anxiety, interpersonal sensitivity, depression, and hostility using a 5-point Likert scale (1 = not at all, 5 = very much). It was adopted from the Symptom Check List (SCL-90-R) from Kim et al. (1978) [32], which is the abbreviated Korean version of the Hopkins Symptom Check List (SCL-90-R: Symptom Check List-90-Revised) originally developed by Derogatis et al. (1976) [33].

### 2.2. Data Collection

The study focused on university students in Gyeongnam, Republic of Korea, who were selected as to represent the young generation. Participants in their twenties are at the peak of awareness of social norms around appearance [34,35], and it is expected to be a time of interest in weight management. A total of 239 undergraduate students from the Department of Food and Nutrition participated in the survey in a classroom by convenient sampling. The survey was conducted using a self-administration method, lasting two weeks. A total of 240 participants initially completed the survey, with 239 producing valid responses.

## 3. Results

### 3.1. Study Participants

Table 3 presents the demographic profiles of the study participants. Of the respondents, 32.2% were male and 67.8% were female. Approximately 21.8% were freshmen, 46.4% were sophomores, 20.9% were juniors, and 2.1% were seniors. Approximately half of the respondents showed a BMI in the normal range (42.7%), 38.9% were in the overweight or obese range, and 13.8% were underweight. The attitudes of the respondents toward their bodies were as follows: dissatisfied (43.5%), neutral (40.6%), satisfied (10.5%), and very dissatisfied (4.6%). In addition, more than half of the respondents responded that they control their weight for self-satisfaction and confidence (59.0%), while others controlled their weight for healthcare (25.1%), a preferred body shape (11.3%), and better interpersonal relationships (1.7%). Even though nearly half of the respondents had a BMI within the normal range, 73.2% of the respondents wanted to lose weight, and 8.4% wanted to gain weight. Approximately 18.4% of respondents wanted to maintain their weight.

### 3.2. Reliability and Validity Analyses

The goodness-of-fit indices were calculated using AMOS 24.0, and validity was verified through a confirmatory factor analysis (CFA). To confirm construct validity, construct reliability (CR) and average variance extracted (AVE) were analyzed. The AVE ranged from 0.456 to 0.940, with most above the limit of 0.5, and CR value ranged from 0.626 to 0.987, with most above the standard of 0.7. Discriminant validity was confirmed by comparing the shared variance and AVE values of all components (Table 4). The AVE values for the constructs of this study are greater than the squared correlation coefficient between the factors, which indicates discriminant validity of the measurement model [36]. Examining the model fit, the chi-square value was 1057.21, df was 596, CMIN/DF was 1.774 (acceptable, as it is <3), and RMSEA was 0.057 (acceptable, as it is <0.08) [37]. The *p*-value was less than 0.001, indicating significance. Therefore, the goodness-of-fit indices of the measurement model signified an appropriate system. Inter-item reliability analysis was performed using SPSS version 27, and Cronbach’s alpha was measured as the extent to which an item is free from random error and can produce consistent results. Cronbach’s alpha ranged from 0.709 to 0.943, above the 0.7 suggested in previous studies [36]. The questionnaire items used in the survey, results of CFA, and Cronbach’s alpha values are shown in Table 2.

In this study, mental health was assessed as a formative construct of a composite factor through five dimensions [38]. Consequently, the average values of the sub-constructs anxiety, obsession-compulsivity, depression, interpersonal sensitivity, and hostility were used as index for the formative construct, which serves as a second-order factor.

### 3.3. Correlation Matrix

Pearson’s coefficient of correlation was analyzed to determine the correlation between the latent variables used in the extended MGB established in this study. All correlation coefficients were lower than 0.8, indicating that there was no problem of multicollinearity. There was no correlation between the latent variables, and there was a significant correlation between the sub-variables of the latent variables.

## 4. Results of Hypothesis Testing

**Figure 2:**
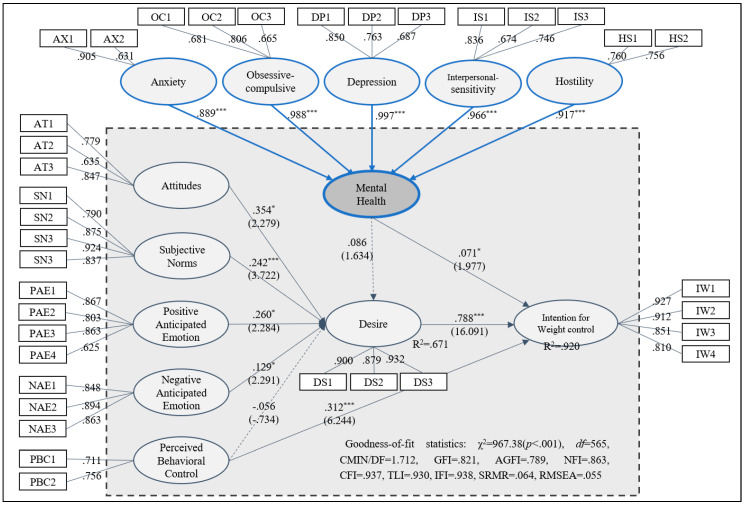
Results of the structural equation model (*N* = 239). Note 1. Blue lines = formative indicators. Note 2. Mental health = second-order factor; attitude, subjective norm, positive anticipated emotion, negative anticipated emotion, perceived behavioral control, desire, and behavior intention = first-order factors. Note 3. * *p* < 0.05; *** *p* < 0.001.

As shown in Table 5 and Table 6, and Figure 2, the nine hypotheses were examined using a structural equation model (SEM). The total model fit of the SEM analysis was adequate (χ^2^/df = 967.38; *p* < 0.001; df = 565; CMIN/DF = 1.712; GFI = 0.821; AGFI = 0.789; NFI = 0.863; CFI = 0.937; TLI = 0.930; IFI = 0.938; SRMR = 0.064; RMSEA = 0.055). Attitude toward weight control (β = 0.548, *p* < 0.05), subjective norm (β = 0.236, *p* < 0.01), positive anticipated emotion toward weight control (β = 0.379, *p* < 0.05), and negative anticipated emotion (β = 0.100, *p* < 0.05) had significant effects on desire. Hypotheses 1, 2, 3, and 4 were supported, while hypothesis 5, representing the association between perceive behavioral control and desire, was rejected (β = −0.067, *p* > 0.05). Perceived behavioral control (β = 0.436, *p* < 0.001) and desire (β = 0.922, *p* < 0.001) had significant effects on intention; thus, hypotheses 6 and 7 were supported. Hypothesis 9 was supported by the positive impact of mental health on intention (β = 0.379, *p* < 0.05), but hypothesis 8, which examined the influence of mental health on desire (β = 0.136, *p* > 0.05), was rejected.

## 5. Discussion

Seven of the nine hypotheses in the study model were supported with a satisfactory fit for the dataset. Our findings highlight the significant influences of psychological variables on DE, suggesting that AT and SN are important factors in predicting DE in the weight control context. This is consistent with the research evidence of TPB on weight reduction behavior [39,40]. Schifter and Ajzen (1985) applied TPB to weight loss among college women and found that AT and SN were significant predictors of intention to lose weight [40]. Similarly, in a study conducted by Mazloomy Mahmoodabad et al. (2018) [39], AT and SN regarding weight loss positively influenced intention to lose weight among adolescents who were overweight or obese. In the study of Kim and Kim (2023), SN from parents or professors significantly influenced the frequency of breakfast consumption among female university students [41]. Several studies revealed AT as the most influential variable for DE among the antecedent variables of desire [42,43].

Our findings revealed PAE and NAE as important determinants in predicting IW in the weight control context. This indicates that the impacts of emotional variables have a strong impact on the desire for weight control among undergraduate students. That is, emotional responses to success or failure in weight control behaviors further enhance voluntary motivation to achieve goals. It appears that individuals are more likely to manage their weight effectively when influenced by emotional factors, with positive anticipated emotions having a more significant impact compared with negative ones. A study by Baranowski et al. (2015) found a negative relationship between ineffective parenting practices toward vegetable consumption and anticipated negative emotional responses of parents to a child’s refusal of vegetables [44]. The extended MGB contributes by incorporating emotional factors to broaden the scope of weight control research, in contrast to the TPB that does not consider emotions. This framework expands the existing knowledge by integrating emotional considerations into weight control strategies and highlights the importance of both rational and emotional factors in weight management. Existing nutrition intervention research predominantly relied on the TPB, which focuses primarily on cognitive variables [45,46].

Our findings revealed that PBC directly influences behavior, rather than indirectly through desire. This indicates that a strong perception of one’s ability to control weight can lead to a strong intention to control weight, even without a corresponding desire. This result is consistent with prior research that established a link between PBC and both exercise intention [47,48] and diet intention [48]. Furthermore, our results indicated that a stronger desire leads to a higher intention, with DE having the most significant impact on the formation of weight control intention. This is consistent with previous research showing that DE plays a critical influential role in the decision-making process related to weight control [44,49,50]. The mediating role of DE on the links between antecedent variables and weight control intention in the extended MGB model indicates that the preceding factors ultimately lead to behavioral intentions through DE in the weight control context. Thus, if considering strategies to enhance desire intensity in a weight control program, nutrition intervention or education programs would be effective.

Our findings highlight the significant and novel role of mental health in weight control intention. Specifically, as mental health improves, the intention to engage in weight control increases. This is consistent with prior research demonstrating a strong association between poor mental health including depressive symptoms, anxiety, and loneliness and various unhealthy behaviors [27,30]. Negative emotions can lead to difficulties in behavioral control such as dysregulated eating [25], which may be attributed to feeling of loss of control [29]. According to Clark et al. (1996), depression and anxiety can impede weight loss success in overweight and obese individuals through the use of food as a means of managing negative emotion [28]. Consequently, lack of motivation and lethargy (the symptoms of depression) can decrease the intention to control weight. This finding is important given the lack of direct empirical evidence confirming the influence of mental health on weight control intention.

Our study proposes a new model that applies MGB [16] to a weight control context and integrates mental health. While the TPB has been highly predictive in explaining health behavior, it was limited to cognitive aspects. The current study enhanced its explanatory power by incorporating both cognitive and affective decision-making processes. The extension of MGB in the weight control context provides a useful framework for health behavior-related academic literature. This study found the importance of affective factors. Thus, an effective intervention can be used to develop emotionally empathetic value-stimulating educational programs. Considering the critical roles of mental health in weight control desire and intention, incorporating mental health consideration into nutrition intervention programs can lead to successful outcomes.

## 6. Conclusions

This study highlights the role of affective factors as a key motivational elements, alongside cognitive aspects emphasized in the TPB theory, by applying the MGB theory to weight control intentions [51]. Furthermore, it enhances its explanatory power in predicting weigh control intention by incorporating mental health into the extended MGB model [52,53]. It provides research evidence that can be helpful in developing an effective weight control intervention program.

## 7. Limitations

While this study has meaningful contribution, there are also some limitations [54,55,56]. The data were collected from university students using convenient sampling; thus, the present findings may not be generalized to different ages. A self-administered questionnaire used in this study may introduce biases such as non-response bias and self-report bias, although it is a convenient and cost-effective survey tool [54,56]. The participants’ sociodemographic factors, such as economic level and geography, were insufficiently investigated. Future research can be improved by conducing with a larger population considering the influence of socio-demographic characteristics (age, BMI, gender, income, marital status) on an individual’s behavioral intention [55]. This study can be improved by comparing the group with prior weigh loss program experience to the non-participating group, thereby investigating the effect of the intervention. Finally, in this study, mental health was assessed using only five constructs. Incorporating additional constructs would enable a more comprehensive and expansive evaluation of overall mental health.

## Figures and Tables

**Table 1:** Outcome measures (*N* = 239).

Variables	Mean (S.D.)
Attitude	5.72 (0.79)
Subjective norm	5.18 (1.23)
Positive anticipated emotion	5.96 (0.83)
Negative anticipated emotion	4.25 (1.49)
Perceived behavior control	4.99 (0.94)
Desire	5.61 (1.17)
Intention for weight control	5.45 (1.19)
Anxiety	1.45 (0.54)
Obsession-compulsivity	1.88 (0.66)
Depression	1.66 (0.66)
Interpersonal sensitivity	1.67 (0.61)
Hostility	1.33 (0.47)

**Table 2:** Confirmatory factor analysis (CFA) and convergent validity.

Measurement Items	B	S.E.	CR	AVE	*Cronbach’s α*
***Attitude (AT)***					
I believe that weight control is beneficial.	0.784	0.377	0.778	0.543	0.799
I believe that managing one’s weight is appealing.	0.636	0.801			
I believe that weight control is necessary.	0.843	0.282			
***Subjective norm (SN)***					
People around me will support my efforts to control my weight.	0.789	0.753	0.853	0.594	0.916
People around me will understand my effort to control my weight.	0.874	0.430			
People around me will agree with my efforts to control my weight.	0.925	0.253			
People around me will recommend weight control.	0.838	0.578			
***Positive anticipated emotion (PAE)***					
If I succeed in controlling my weight, I will be glad.	0.868	0.221	0.873	0.635	0.866
If I succeed in controlling my weight, I will be satisfied.	0.799	0.342			
If I succeed in controlling my weight, I will be happy.	0.864	0.240			
If I succeed in controlling my weight, I will be proud.	0.629	0.652			
***Negative anticipated emotion (NAE)***					
If I fail to control my weight, I will be angry.	0.846	0.843	0.766	0.522	0.902
If I fail to control my weight, I will be disappointed.	0.894	0.566			
If I fail to control my weight, I will be sad.	0.866	0.664			
***Perceived behavioral control (PBC)***					
I am capable of controlling my weight.	0.721	0.652	0.626	0.456	0.709
I am confident that I can control my weight.	0.764	0.664			
***Desire (DE)***					
I would like to control my weight.	0.904	0.294	0.913	0.724	0.943
I desire to control my weight.	0.876	0.377			
I hope to control my weight.	0.923	0.235			
I want to control my weight.	0.888	0.323			
***Intention for weight control (IW)***					
I intend to control my weight in the near future.	0.927	0.265	0.890	0.670	0.927
I am planning to control my weight in the near future.	0.911	0.364			
I will try to succeed in controlling my weight.	0.849	0.347			
I will invest time in controlling my weight in the near future.	0.814	0.540			
Mental health (MH) *					
Anxiety	0.889	0.138	0.987	0.940	0.922
Obsession-compulsivity	0.988	0.013			
Depression	0.997	0.004			
Interpersonal sensitivity	0.966	0.051			
Hostility	0.917	0.083			

Note 1. Blue = standardized regression coefficient; S.E. = standard error; CR = construct reliability; AVE = average variance extracted. Note 2. Goodness-of-fit statistics: χ^2^ = 1057.21 (*p <* 0.001), *df* = 596, CMIN/DF = 1.774, GFI = 0.811, AGFI = 0.777, NFI = 0.859, CFI = 0.932, TLI = 0.924, IFI = 0.933, SRMR = 0.064, RMSEA = 0.057. Note 3. * A second-order factor.

**Table 3:** Demographic characteristics of the respondents (*N* = 239).

Variables	Classification	*N* (%)
Gender	Male	77 (32.2)
	Female	162 (67.8)
Grade	First-year student	52 (21.8)
	Second-year student	111 (46.4)
	Third-year student	50 (20.9)
	Fourth-year student	5 (2.1)
	Other	21 (8.8)
BMI	Underweight	33 (13.8)
	Normal	102 (42.7)
	Overweight or obese	93 (38.9)
	Other	11 (4.6)
Satisfaction toward body shape	Not satisfied at all	11 (4.6)
	Dissatisfied	104 (43.5)
	Neutral	97 (40.6)
	Satisfied	25 (10.5)
	Very satisfied	2 (0.8)
Reason for weight control	For a popularly preferred body shape	27 (11.3)
	For health	60 (25.1)
	For better interpersonal relationships	4 (1.7)
	For self-satisfaction and confidence	141 (59.0)
	Other	7 (2.9)
Type of desired weight control	Weight loss	175 (73.2)
	Weight maintenance	44 (18.4)
	Weight gain	20 (8.4)

**Table 4:** Discriminant validity and correlation estimates (*N* = 239).

	AT	SN	PAE	NAE	PBC	DE	IW	MH
AT	0.543							
SN	0.627 **	0.59						
PAE	0.830 **	0.507 **	0.64					
NAE	0.427 **	0.429 **	0.390 **	0.52				
PBC	0.571 **	0.338 **	0.437 **	0.172	0.46			
DE	0.746 **	0.624 **	0.721 **	0.490 **	0.386 **	0.72		
IW	0.799 **	0.644 **	0.668 **	0.533 **	0.570 **	0.935 **	0.67	
MH	0.087	0.049	0.199 **	0.226 **	−0.180 *	0.226 **	0.198	0.94

Note 1. * *p* < 0.05; ** *p* < 0.01. Note 2. (1) Correlation coefficient; (2) AVE = average variance extracted. Note 3. AT = attitude; SN = subjective norm; PAE = positive anticipated emotion; NAE = negative anticipated emotion; PBC = perceived behavioral control; DE = desire; IW= intention for weight control; MH = mental health.

**Table 5:** Path model results (*N* = 239).

Hypothesis	Path	*β*	S.E.	C.R.	*p*	B	Results
H1	AT→DE	0.548	0.241	2.279	0.023	0.354	Supported
H2	SN→DE	0.236	0.063	3.722	0.001	0.242	Supported
H3	PAE→DE	0.379	0.166	2.284	0.022	0.260	Supported
H4	NAE→DE	0.100	0.044	2.291	0.022	0.129	Supported
H5	PBC→DE	−0.067	0.092	−0.734	0.463	−0.056	Rejected
H6	PBC→IW	0.436	0.070	6.244	0.001	0.312	Supported
H7	DE→IW	0.922	0.057	16.091	0.001	0.788	Supported
H8	MH→DE	0.136	0.083	1.634	0.102	0.086	Rejected
H9	MH→IW	0.132	0.067	1.977	0.048	0.071	Supported

Note 1. AT = attitude; SN = subjective norm; PAE = positive anticipated emotion; NAE = negative anticipated emotion; PBC = perceived behavioral control; DE = desire; IW= intention for weight control; MH = mental health.

**Table 6:** Indirect effect on latent variables.

		B	*p*
AT	→IW	0.279	0.057
SN		0.190	0.002
PAE		0.205	0.066
NAE		0.101	0.028
PBC		−0.044	0.490
MH		0.068	0.134

Note 1. AT = attitude; SN = subjective norm; PAE = positive anticipated emotion; NAE = negative anticipated emotion; PBC = perceived behavioral control; DE = desire; IW= intention for weight control; MH = mental health.

## Data Availability

The data that support the findings of this study are available upon reasonable request.
